# Genetic factors associated with thymic tumors in patients with MEN1: a nested case-control study in the GTE/AFCE cohort

**DOI:** 10.1210/jendso/bvag159

**Published:** 2026-07-21

**Authors:** Antoine Journé, Pierre Goudet, Amadou-Khalilou Sow, Sandrine Daniel, Annie Costa, Sophie Nambot, Côme Lepage, Christine Binquet

**Affiliations:** Université Bourgogne Europe, CHU Dijon Bourgogne, Centre d’Investigation Clinique, Module épidémiologie Clinique, INSERM, CIC1432, Côte d'Or, 21000 Dijon, France; Université Bourgogne Europe, INSERM, UMR1231, équipe EPICAD, Côte d'Or, 21000 Dijon, France; Université Bourgogne Europe, CHU Dijon Bourgogne, Centre d’Investigation Clinique, Module épidémiologie Clinique, INSERM, CIC1432, Côte d'Or, 21000 Dijon, France; Université Bourgogne Europe, INSERM, UMR1231, équipe EPICAD, Côte d'Or, 21000 Dijon, France; Université Bourgogne Europe, CHU Dijon Bourgogne, Centre d’Investigation Clinique, Module épidémiologie Clinique, INSERM, CIC1432, Côte d'Or, 21000 Dijon, France; Université Bourgogne Europe, CHU Dijon Bourgogne, Centre d’Investigation Clinique, Module épidémiologie Clinique, INSERM, CIC1432, Côte d'Or, 21000 Dijon, France; Université Bourgogne Europe, CHU Dijon Bourgogne, Centre d’Investigation Clinique, Module épidémiologie Clinique, INSERM, CIC1432, Côte d'Or, 21000 Dijon, France; Université Bourgogne Europe, INSERM, UMR1231, équipe EPICAD, Côte d'Or, 21000 Dijon, France; Université Bourgogne Europe, CHU Dijon Bourgogne, Inserm, CTM UMR1231, équipe GAD, FHU TRANSLAD, Centre de Génétique, Centre de Référence Anomalies du Développement et Syndromes Malformatifs, Côte d'Or, 21000 Dijon, France; Service d'Oncogénétique, Centre Georges-François Leclerc, UNICANCER, Côte d'Or, 21000 Dijon, France; Université Bourgogne Europe, INSERM, UMR1231, équipe EPICAD, Côte d'Or, 21000 Dijon, France; Service D’hépato-Gastro-Entérologie, CHU Dijon Bourgogne, Côte d'Or, 21000 Dijon, France; Université Bourgogne Europe, CHU Dijon Bourgogne, Centre d’Investigation Clinique, Module épidémiologie Clinique, INSERM, CIC1432, Côte d'Or, 21000 Dijon, France; Université Bourgogne Europe, INSERM, UMR1231, équipe EPICAD, Côte d'Or, 21000 Dijon, France

**Keywords:** genetic factor, thymic tumor, MEN1

## Abstract

**Context:**

Multiple endocrine neoplasia type 1 (MEN1) is a rare autosomal dominant syndrome characterized by tumors in multiple endocrine tissues. Its management is challenging because of its unpredictable course, particularly regarding thymic tumors, whose prognosis remains poor.

**Objective:**

To identify the genetic factors associated with the development of thymic tumors in patients with MEN1.

**Design:**

Nested case-control study in the Groupe d’étude des Tumeurs Endocrines and of the Association Francophone de Chirurgie Endocrinnienne cohort (>1600 patients included from the 1990s through 2024).

**Setting:**

Ambulatory and hospitalized care in referral centers.

**Patients:**

All men with a confirmed thymic tumor in the Groupe d’étude des Tumeurs Endocrines/Association Francophone de Chirurgie Endocrinnienne cohort were selected and matched to 2 nearest controls without thymic tumors, with a diagnosis of MEN1 identified at the same period (<1990; 1990-2000; ≥2000) and with an age at their last follow-up at least equal to the age of their paired case at the time of thymic tumor discovery.

**Main Outcome Measure:**

Development of thymic tumor.

**Results:**

Fifty-two cases and 104 controls were included. Mean age at diagnosis of thymic tumor was 44 (SD = 11). Nonsense mutations were associated with a higher risk of thymic tumors (OR = 3.77; 95% CI, 1.36-10.50) than men with frameshift mutations. Among cases, the median age of onset of thymic tumor was almost identical between patients with a nonsense mutation and those without (43.8 vs 43.2 years, respectively).

**Conclusion:**

Men carrying nonsense MEN1 mutations may have an increased risk of thymic tumors and could benefit from enhanced thymic surveillance.

Multiple endocrine neoplasia type 1 (MEN1) is a rare autosomal dominant syndrome resulting from mutations inactivating the MEN1 gene (11q13). This gene encodes a tumor suppressor protein called menin [1]. Its prevalence varies between 1/10 000 and 1/30 000 [1]. This disease has a high degree of penetrance (>80%) [2] and is characterized by the development of tumors in various endocrine tissues. Certain types of tumors are particularly common in this condition and are referred to as cardinal. These include parathyroid tumors (95%), pancreatic tumors (40%), and pituitary tumors (30%). But other, less frequent disorders are also characteristic of this disease and may be marred by a poor prognosis, such as tumors of the thymus [3-5], with 10-year survival of 33.3% [6], and/or with a significant metastatic potential, such as gastrinomas [7]. The adrenal cortex and central nervous system can also be affected, as can skin tissue [8]. Most tumors produce and secrete peptide hormones (in the case of parathyroid or pituitary involvement, but also in the duodeno-pancreas in the case of insulinoma, gastrinoma, VIPoma, glucagonoma, or PPoma, for example). These hypersecretions are responsible for specific clinical syndromes [9] and, if left uncontrolled, can affect patients’ prognosis and quality of life [10]. The first clinical manifestations usually occur around the age of 30 or 40 years [11, 12], but may also occur earlier [13]. Although MEN1 is generally slow and benign, it is estimated that the life expectancy of patients with this disease is 15 years shorter than that of the general French population [14].

A diagnosis of MEN1 can be made based on clinical, familial, or genetic criteria. Thus, a clinical MEN1 diagnosis could be confirmed if patients had 2 or more MEN1-associated tumors. A familial form of MEN1 was retained if patients had at least 1 MEN1-associated tumor in addition to a first-degree relative with a confirmed diagnosis of MEN1. Finally, a genetic diagnosis was considered if a germline MEN1 mutation was identified [8].

The complexity of this disease has led several groups to set up cohorts of patients with this pathology. This is how the MEN1 cohort of the Groupe d'Etude des Tumeurs Endocrines (GTE) and the Association Française de Chirurgie endocrinienne (AFCE) was initiated in the 1990s. This cohort, coordinated in Dijon, is now the largest cohort worldwide, with >1600 patients included to date.

One of the main difficulties in supporting patients with MEN1 is the largely unpredictable prognosis of the disease. Indeed, there is no clear genotype-phenotype correlation [15-17] and in particular, the genetic factors leading to the development of thymic tumors, which are still debated [18-22]. For this reason, we took advantage of the large size of the GTE/AFCE cohort to attempt to identify the factors associated with the development of thymic tumors in patients with MEN1 in France.

## Materials and methods

Our study relied on a case-control study nested within the GTE/AFCE MEN1 cohort. Briefly, this cohort relies on the GTE and AFCE networks for MEN1 [23], created in February 1991; it includes 22 reference clinical centers in France as well as the 4 genetics departments responsible for diagnosing MEN1. It includes symptomatic individuals with a confirmed diagnosis of MEN1 living in France. According to international criteria [24], and as reported elsewhere [7] the following criteria were used to confirm MEN1 disease: (1) patients with a MEN1 mutation and having with at least 1 MEN1-related lesion, (2) patients belonging to an already known MEN1 family in which at least 1 first-degree relative has been affected and who had at least 1 MEN1-related lesion, and (3) patients without positive genetic testing and without a family background, with at least 2 of the 3 major MEN1 lesions (primary hyperparathyroidism, duodeno-pancreatic tumors, and pituitary tumors). Patients who presented only 2 major lesions were considered with caution because these associations may occur randomly in the general population. Patients with a single-organ genetic endocrine disease linked to another genetic syndrome (familial pituitary disease, familial hyperparathyroidism, etc.) are not included in this cohort.

For each patient, a case report form is filled in and checked by the physician coordinating the cohort (P.G.) and regularly updated with the help of the referent physician. Genetic testing for *MEN1* is performed by Sanger sequencing, multiplex ligation–dependent probe amplification analysis, or next-generation sequencing on leukocyte DNA from patients reviewed in 1 of the laboratories of the TENGEN network. Data are gathered in a secured database at the Dijon Clinical Investigation Center (INSERM CIC1432) and monitored regularly. The database includes the date of diagnosis, the tumor characteristics (localization, size, histology), main genetic data (genetic test performed or not, date of the test, family reference, type of mutation, location of the mutation), treatments (type of surgery, surgical outcome), and follow-up and survival status at the endpoint. Written and signed informed consent is obtained from patients following the French rules regarding observational cohort. The MEN1 cohort was approved by the Institutional Review Board CPP Sud-Est V (2018-A0192847), the CCTIRS (Consultative Committee on Treatment of Information in Health Research, file number 12.364), and the CNIL (National Committee for Data Protection, authorization number DR 2013-348, tacitly renewed in May 2024 following application no. 924065.).

Because thymic tumors occur mainly in men [6], all men with a confirmed thymic tumor in the GTE/AFCE cohort were selected and matched to the 2 nearest controls without thymic tumors [25], with a diagnosis of MEN1 identified at the same period (<1990; 1990-2000; ≥2000) and with an age at their last follow-up at least equal to the age of their paired case at the time of thymic tumor discovery. To be selected for the study, cases and controls had to have available data on their genetic characteristics and family history as well as on their outcomes after MEN1 diagnosis. Overall, 52 men with a confirmed thymic tumor and were identified in the GTE/AFCE cohort. With this number of cases, a target power of 80%, a significant level of 0.05, with 2 controls by case, we expected to be able to evidence odds ratio of 2.8 between the type or location of variants in the MEN1 gene presented in around 25% of the MEN1 population and thymic tumor occurrence.

To investigate factors associated with thymic tumors, a conditional logistic regression model was used. First, variables related to genetic characteristics associated with a *P* value equal to or lower than .20 in bivariable models were selected for inclusion in the multivariable analysis. Then, the multivariable conditional logistic regression was used to estimate the adjusted association between type of mutations, and then between each of their location, and thymic tumor occurrence. We chose the type of mutation with the largest number of individuals as a reference category in order to stabilize the model [26]. Associations are presented using odds ratios (OR) and their 95% CIs. Discriminant ability of the model was evaluated using weighted area under the curve specific to matched case-control studies [27, 28]. A sensitivity analysis following the same strategy was performed using a mixed conditional logistic approach with a family-related random effect. The area under the curve of both modeling strategies were compared to select the most appropriate one. In case of these being tied, the most parsimonious model was selected. Statistical analyses were performed using R software version 2024.12.0.

## Results

**Figure 1:**
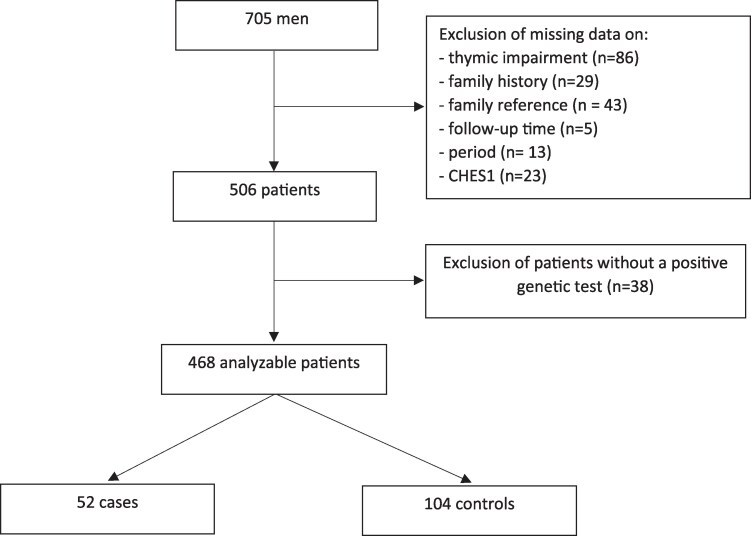
Selection process of the cases ans controls for the study in the GTE/AFCE cohort (1990-2024). AFCE, Association Française de Chirurgie endocrinienne; GTE, Groupe d'Etude des Tumeurs Endocrines.

After excluding patients with missing data on their outcome after MEN1 diagnosis and particularly on thymic involvement, but also on their family history and genetic characteristics, 476 patients remained for our study (show in Fig. 1). Among them, 52 men had a thymic tumor and 104 paired controls were selected. Mean age at diagnosis of thymic tumor was 44 years (SD = 11; show in Table 1). They were mainly diagnosed with MEN1 mainly after 1990 (88%). In bivariate analyses, cases and controls did not differ significantly except concerning their age at MEN1 diagnosis (38 years ± 13 for cases vs 32 ± 15 for controls; *P* = .002).

**Table 1:** Medical history, type, and location of mutations in MEN1 included patients and univariable analysis with conditional logistic regressions (GTE/AFCE cohort 1990-2024)

Variable*^a^*	Overall (n = 156)	Controls (n = 104)	Cases (n = 52)	Unadjusted OR (95% CI)*^b^*	*P* value*^b^*
Age at diagnosis of thymic tumor	/	/	44 (11)	/	/
Age at last reporting date	/	45 (16)	/	/	/
Age at diagnosis of MEN1	34 (15)	32 (15)	38 (13)	1.06 (1.02-1.10)	.002
Diagnosis period for MEN1					.49
< 1990	22 (14%)	16 (15%)	6 (12%)	Reference	Reference
[1990-2000]	56 (36%)	35 (34%)	21 (40%)	2.53 (0.46-13.89)	.29
> 2000	78 (50%)	53 (51%)	25 (48%)	2.00 (0.35-11.39)	.44
Parathyroid tumor	143 (92%)	98 (94%)	45 (87%)	0.39 (0.12-1.25)	.11
Nonsecreting tumor	57 (37%)	36 (35%)	21 (40%)	1.27 (0.65-2.47)	.49
Gastrinoma	37 (24%)	25 (24%)	12 (23%)	0.94 (0.42-2.14)	.89
Insulinoma	12 (7.7%)	10 (9.6%)	2 (3.8%)	0.40 (0.09-1.83)	.24
Pituitary tumor	56 (36%)	41 (39%)	15 (29%)	0.64 (0.32-1.29)	.21
Adrenal tumor	45 (29%)	31 (30%)	14 (27%)	0.88 (0.44-1.78)	.72
Family history of MEN1	133 (85%)	91 (88%)	42 (81%)	0.59 (0.23-1.49)	.26
Nonsense	35 (22%)	18 (17%)	17 (33%)	2.45 (1.09-5.54)	.03
Frameshift	55 (35%)	42 (40%)	13 (25%)	0.51 (0.25-1.06)	.07
Splicing	20 (13%)	11 (11%)	9 (17%)	1.68 (0.68-4.18)	.26
Missense	23 (15%)	20 (19%)	3 (5.8%)	0.27 (0.08-0.95)	.04
In-frame	7 (4.5%)	3 (2.9%)	4 (7.7%)	5.26 (0.55-50.02)	.14
Other	13 (8.3%)	7 (6.7%)	6 (12%)	1.80 (0.57-5.69)	.31
Exon 2	20 (13%)	14 (13%)	6 (12%)	0.84 (0.31-2.30)	.74
Exon 3	17 (11%)	13 (13%)	4 (7.7%)	0.60 (0.19-1.89)	.38
Exon 4	8 (5.1%)	6 (5.8%)	2 (3.8%)	0.67 (0.13-3.30)	.62
Exon 5	5 (3.2%)	5 (4.8%)	0 (0%)	—	—
Exon 6	1 (0.6%)	0 (0%)	1 (1.9%)	—	—
Exon 7	8 (5.1%)	4 (3.8%)	4 (7.7%)	2.26 (0.49-10.42)	.30
Exon 8	2 (1.3%)	2 (1.9%)	0 (0%)	—	—
Exon 9	20 (13%)	15 (14%)	5 (9.6%)	0.64 (0.22-1.84)	.41
Exon 10	41 (26%)	26 (25%)	15 (29%)	1.20 (0.59-2.45)	.62
Intron 2	3 (1.9%)	1 (1.0%)	2 (3.8%)	4 (0.36-44.11)	.26
Intron 3	1 (0.6%)	1 (1.0%)	0 (0%)	—	—
Intron 4	15 (9.6%)	8 (7.7%)	7 (13%)	1.83 (0.63-5.29)	.26
Intron 5	0 (0%)	0 (0%)	0 (0%)	—	—
Intron 6	0 (0%)	0 (0%)	0 (0%)	—	—
Intron 7	0 (0%)	0 (0%)	0 (0%)	—	—
Intron 8	0 (0%)	0 (0%)	0 (0%)	—	—
Intron 9	1 (0.6%)	1 (1.0%)	0 (0%)	—	—
JunD	58 (37%)	40 (38%)	18 (35%)	0.86 (0.44-1.67)	.65
CHES1	64 (41%)	42 (40%)	22 (42%)	1.08 (0.56-2.08)	.82

Abbreviations: AFCE, Association Française de Chirurgie endocrinienne; GTE, Groupe d'Etude des Tumeurs Endocrines; MEN1, Multiple Endocrine Neoplasia Type 1; OR, odds ratio; /, not applicable due to matching; -, calculation impossible due to null categories.

^*a*^Continuous variables described as mean (±SD); categorical variables described as n (%).

^*b*^Conditional logistic regression.

**Figure 2:**
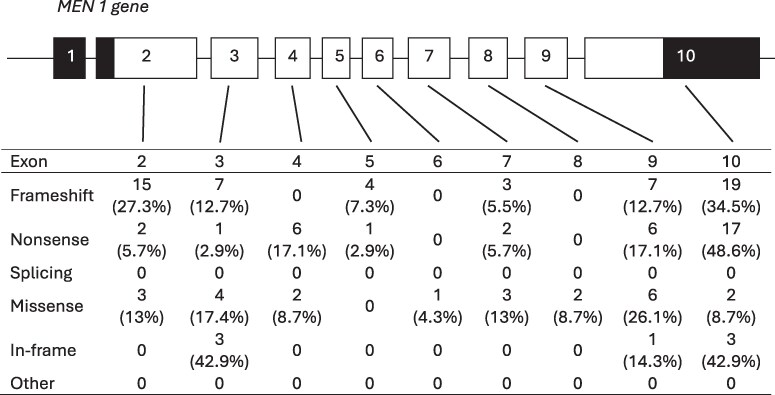
Distribution of different types of mutations according to exons in patients with MEN1 (GTE/AFCE cohort 1990-2024). From Molina-Céspedes P *Clin Case Rep,* 2023; 11(3). AFCE, Association Française de Chirurgie endocrinienne; GTE, Groupe d'Etude des Tumeurs Endocrines.

If we look at the distribution of different types of mutations according to their location in the different exons, we can see that nonsense mutations are mainly distributed in exon 10, whereas frameshift mutations appear mainly in exons 2 and 3 (shown in Fig. 2).

Both modeling strategies led to similar results and discriminant ability, leading to retain the results of the conditional logistic regressions as the final results of the study. In multivariable conditional logistic regressions, nonsense mutations were associated with a higher risk of thymic tumors (OR = 3.77; 95% CI, 1.36-10.50; show in Table 2) than men with a frameshift mutation. Among cases, the median age of onset of thymic tumor was almost identical between patients with a nonsense mutation and those without (43.8 vs 43.2 years, respectively). Splicing mutations appeared to be marginally associated with a higher risk of thymic tumors (OR = 2.98; 95% CI, 0.99-8.92) compared to patients with frameshift mutations. No other significant association were found according to the mutations’ location.

**Table 2:** Associations with thymic tumors in patients with MEN1, multivariable conditional logistic regression (GTE/AFCE cohort 1990-2024)

Variable	Overall*^a^*	Adjusted OR (95%-CI)*^b^*	*P* value*^b^*
Frameshift	55 (35%)	Reference	Reference
Nonsense	35 (22%)	3.77 (1.36-10.50)	.01
Missense	23 (15%)	0.49 (0.13-1.90)	.30
Splicing	20 (13%)	2.98 (0.99-8.92)	.05
Other	13 (8.3%)	2.92 (0.83-10.30)	.10
In-frame	7 (4.5%)	4.60 (0.47-44.98)	.19

^*a*^n (%).

^*b*^Conditional logistic regression.

Abbreviations: AFCE, Association Française de Chirurgie endocrinienne; GTE, Groupe d'Etude des Tumeurs Endocrines; OR, odds ratio.

Weighted area under the curve: 0.69 (0.58-0.74).

## Discussion

Our study evidenced that nonsense mutations in men with MEN1 are associated with a higher risk of thymic tumors occurrence. Nevertheless, these results concerned only men. Indeed, the potentially different genotype-phenotype relationships between women and men [17, 29] led us to consider a stratified case-control study, but the number of women with thymic tumors in our cohort was too low (n = 5) to allow a sufficient power in this group. This point constitutes a limitation and caution should be exercised when extrapolating the results to women. In addition, we cannot exclude a lack of power of our analyses as with our sample size we could only detect ORs above 2.88. Thus, our ability to identify moderate associations particularly for splicing, in-frame, and exon-specific effects was limited and our estimates are framed by wide CIs. However, to the best of our knowledge, our study is the largest providing estimations of the association between genetic characteristics and thymic tumor occurrence and the only one accounting for intrafamily correlation. In addition, all the medical files of included patients were thoroughly reviewed by a medical expert (P.G.), which first ensured data quality.

Furthermore, we observed a significant difference between cases and controls in terms of age at the time of diagnosis of MEN1 syndrome. This could potentially introduce a surveillance bias. However, as the age at diagnosis was younger among the controls, surveillance began earlier for them than for the cases. Consequently, this bias would likely have attenuated the observed differences rather than amplifying them. Moreover, controls were matched with an age at their last follow-up at least equal to the age of their paired case at the time of thymic tumor discovery, so they have the same follow-up duration. Conversely, even if cases and controls were matched on the period of MEN1 diagnosis, as the periods are quite long, we cannot completely rule out differences in practices.

Few data on genetic characteristics that may favor thymic tumor occurrence are available in the literature, but our results are in line with them. Indeed, Lim et al studied 22 separate MEN1 families with thymic carcinoids and found that all but 2 (91%) had mutations coding for a truncated protein (frameshift, splice, and nonsense), suggesting a high frequency of truncating mutations in MEN1-related thymic carcinoids [18]. In addition, Mandl et al observed also a majority of protein-truncating mutations (85%) in 14 patients (4.8%) with thymic tumors identified in a retrospective chart review of 294 patients with MEN1 germline mutations [20]. Both nonsense and frameshift variants encode truncated proteins, but we showed that these mutations are associated with different risks of thymic tumors. One possible explanation for this difference could be the fact that nonsense mutations introduce a premature stop codon into the MEN1 gene sequence, resulting in the production of a truncated menin protein that is often unstable and rapidly degraded, and a complete or near-complete loss of menin function. Because menin plays a key role in cell cycle regulation, DNA repair, and genomic stability [30], a complete loss of its function (as in nonsense mutations) could promote greater genomic instability in the thymus. This instability would make the cell more vulnerable to a second tumorigenic event, which appears to be systematic in thymic tumors [20]. Furthermore, frameshift variations, with a shift in the reading frame, generally result in a truncated and dysfunctional menin protein, but sometimes with residual protein residues or neoproteins. Some of these abnormal proteins may retain partial activity or interfere differently with menin's cellular partners and thus may less often lead to the development of a thymic tumor.

Our study was not designed to formally evaluate the incremental predictive value of genetic subtype beyond established clinical factors, and we could not develop a multivariable model incorporating both clinical and genetic factors because of the limited number of events. Therefore, the immediate clinical utility of incorporating mutation subtype into routine risk prediction remains uncertain. Nevertheless, we accounted for known factors by stratifying our analyses by sex, matching patients according to the period of MEN1 diagnosis and age at the time of thymic tumor diagnosis or age at last known follow-up, and accounting for family history using a mixed-effects model. Thus, our findings may provide additional information for risk prediction and identifying a subgroup of men with a potentially higher risk could help refine surveillance strategies.

In conclusion, this study showed that nonsense mutations are associated with a higher risk of developing thymic tumors in men with MEN1 and provides new insights into predicting the risk of thymic tumors in this population. We suggest that men carrying nonsense MEN1 mutations may benefit from enhanced thymic surveillance. However, these findings should not be extrapolated to women and must be interpreted cautiously given the limited sample size.

## Data Availability

Some or all datasets generated during and/or analyzed during the current study are not publicly available but are available from the corresponding author on reasonable request.

## Abbreviations

AFCE: Association Française de Chirurgie endocrinienne
GTE: Groupe d'Etude des Tumeurs Endocrines
MEN1: multiple endocrine neoplasia type 1
OR: odds ratio
